# Ginseng and Anticancer Drug Combination to Improve Cancer Chemotherapy: A Critical Review

**DOI:** 10.1155/2014/168940

**Published:** 2014-04-30

**Authors:** Shihong Chen, Zhijun Wang, Ying Huang, Stephen A. O'Barr, Rebecca A. Wong, Steven Yeung, Moses Sing Sum Chow

**Affiliations:** ^1^Department of Pharmacy, Xiamen Medical College, Xiamen, Fujian 361008, China; ^2^Center for Advancement of Drug Research and Evaluation, College of Pharmacy, Western University of Health Sciences, Pomona, CA 91766, USA; ^3^Department of Pharmaceutical Sciences, College of Pharmacy, Western University of Health Sciences, Pomona, CA 91766, USA

## Abstract

Ginseng, a well-known herb, is often used in combination with anticancer drugs to enhance chemotherapy. Its wide usage as well as many documentations are often cited to support its clinical benefit of such combination therapy. However the literature based on objective evidence to make such recommendation is still lacking. The present review critically evaluated relevant studies reported in English and Chinese literature on such combination. Based on our review, we found good evidence from *in vitro* and *in vivo* animal studies showing enhanced antitumor effect when ginseng is used in combination with some anticancer drugs. However, there is insufficient clinical evidence of such benefit as very few clinical studies are available. Future research should focus on clinically relevant studies of such combination to validate the utility of ginseng in cancer.

## 1. Introduction 

The concept of herb-herb or herb-drug combination to enhance therapeutic benefit has been utilized and practiced in China according to Chinese medicine principles for more than 2000 years [1]. Recently, herbs in combinations with anticancer drugs have been found to be capable of resensitizing the chemoresistance developed from repeated use of the anticancer drug [2]. Thus, the use of herb-drug combination to enhance therapeutic effect is of great interest, especially in cancer chemotherapy.

Among many herbs touted to improve cancer treatment, none has probably enjoyed as much worldwide reputation and interest as ginseng. Ginseng is widely used and is included in the pharmacopoeias in China, Japan, Germany, France, Austria, and the United Kingdom. In Asian countries and Western Europe, it is widely available as an over-the-counter drug and also commonly used as an adjuvant for cancer therapy [3, 4]. In the US, ginseng is one of the most frequently purchased herbs; it is available and used as a dietary or an herbal supplement but not as a drug approved by the Food and Drug Administration [5]. It is consumed regularly by more than 6 million Americans [6], as the second top-selling herbal supplement (US $62 million in annual sales in 2000 and about US $83 million in 2010) [7, 8]. In 2002, a national survey of men and women in the US has estimated that 4-5% of those aged 45–64 years had used ginseng [9].

Although ginseng consumption is not limited for in cancer patients, its benefit in cancer appears to be well accepted. Common reasons for the use of ginseng by cancer patients are to improve clinical outcomes, enhance quality of life, treat cancer-related symptoms, reduce adverse effects of chemotherapy, and potentially enhance the effects of chemotherapeutic agents. In addition, ginseng may exert a chemopreventive action: an epidemiological study has shown that patients taking ginseng had a 50% lower risk of cancer recurrence compared to patients not taking ginseng [10].

In view of its wide usage and potential benefit when used in combination with anticancer drugs, the present paper intends to critically review the evidence of such benefit as well as potential mechanisms involved. Although numerous reviews on ginseng-drug interaction have been already published [47, 48], no article has critically reviewed ginseng-anticancer drug combination for improvement of chemotherapy.

Both English and Chinese publications on ginseng and anticancer drug combination to improve cancer chemotherapy were searched from the Medline database (1990~2013) and China Academic Journals Database (1983~2013), respectively. All articles from* in vitro*,* in vivo* animal models, and human studies on the combination of ginseng or its active components with chemotherapeutic agent for anticancer effect were included. Because there are several types of ginseng with different active components, their general properties are briefly discussed to provide relevant background information before reviewing the specific combination in order to provide better understanding of the rationale of such combination.

## 2. Different Types of Ginseng and Its Preparation

Ginseng is a perennial herb that belongs to the Araliaceae family and* Panax *genus [49]. The root is the preferred part of the plant due to the presence of active components (see below), and the species most commonly used are* Panax ginseng* C.A. Meyer (Asian ginseng or Korean ginseng) and* Panax quinquefolius *(American ginseng).* Panax ginseng* C.A. Meyer is usually cultivated in China and Korea and has been used as a medicinal herb in China, Japan, and Korea for thousands of years. Its commonly claimed health benefits include immunity enhancement, stress relief, and prevention of aging.* Panax quinquefolius,* originally grown in United States and Canada, has been used by Native Americans for hundreds of years. So far, majority of research on ginseng has been on* Panax ginseng *C.A. Meyer [5, 17].

*Panax ginseng* C.A. Meyer is usually harvested after 4 to 6 years of cultivation and is classified into three types based on processing methods: (1) fresh (less than 4 years old, consumed in its fresh state), (2) white ginseng (4 to 6 years old, typically air or oven dried after peeling), (3) red ginseng (6 years old, steamed prior to drying, without peeling). These processing methods are intended to improve efficacy, safety, and preservation [50]. Recently, a new heat-processed ginseng, called Sun ginseng (SG), has been prepared by steaming with white ginseng at high temperature and pressure. Sun ginseng has been reported to contain more unique ginsenosides than the red ginseng. A preparation containing Sun ginseng extract with specific standardization is now available as functional food in Korea [21, 22, 24].

Many ginseng products are available on the market as fresh slices, juice, extract (tincture or boiled), powder, tea, tablet, capsule, and other forms. Two-year-old fresh ginseng is also used as an ingredient in Korean chicken-ginseng soup (boiled chicken with young ginseng root),* samketang *[51]. The traditional Chinese ginseng preparation widely used clinically in China is Shengmai which consists of red ginseng, lilyturf root, and magnolia vine fruit [28, 40].

The quality of ginseng is believed to vary with the age at harvest. When ginseng is harvested at the time of 5 to 6 years, it is considered the “best” with ginsenoside content at its highest [52]. According to several laboratory investigations, the quality of commercially available ginseng products can vary considerably. Negative trial results may be due to poor product quality rather than lack of efficacy [35]. Thus, evaluation of study results must take product quality control into consideration.

## 3. Active Components of Ginseng Relevant to Anticancer Effect

Ginseng contains various active components including ginsenosides, polysaccharides, flavonoids, volatile oils, amino acid, and vitamins. Of these active components, ginsenosides and ginseng polysaccharides appear to be responsible for the anticancer effect [8].

Ginsenosides are the main pharmacologically active ingredients responsible for the four major actions of ginseng: vasorelaxation, antioxidation, anti-inflammation, and anticancer effect. Ginsenosides, being amphipathic in nature, are steroidal saponins that contain four transring rigid steroid skeleton. They differ from each other mainly by the number, type, and location of their sugar moieties. Thus far, more than 40 different ginsenosides have been identified and isolated. Ginsenosides can be classified into three groups based on the chemical structure of aglycones: (1) protopanaxadiol group (PPD) or diols, for example, Rb1, Rb2, Rb3, Rc, Rd, Rg3, and Rh2; (2) protopanaxatriol group (PPT) or triols, for example, Re, Rf, Rg1, Rg2, and Rh1; (3) oleanane group: only Ro (0.6% of all ginsenosides) [53, 54]. The total percentage of ginsenosides (w/w) can vary from 1.9% to 8.1% in ginseng root preparations [17]. Red ginseng can possess higher activity than white ginseng, due to the presence of unique ginsenosides (Rg3, Rg5, Rg6, Rh2, Rh3, Rh4, Rs3, and F4) produced during steaming method [15, 18, 24]. The relative amounts of ginsenosides may also be used to differentiate* Panax* species. For example, American ginseng has little or no Rf, and* Panax* ginseng has higher levels of Rg1 but lower levels of Rb1 (or higher ratio of Rg1/Rb1) compared to those of American ginseng [5, 49, 55, 56]. Ginsenosides are also used as marker compounds for ginseng quality control, of which Rg1, Rc, Rd, Re, Rb1, and Rb2 are quantitatively the most important and prevalent. According to a Ginseng Evaluation Program led by the American Botanical Council of Austin, Texas, Rb1, Rb2, Rc, Rd, Re, and Rg1 account for >90% of the total ginsenoside content of the* Panax* ginseng root, whereas, Rb1, Rb3, Rc, Rd, Re, and Rg1 make up more than 70% of total ginsenoside content in American ginseng [8]. Each ginsenoside may differ in pharmacology and mechanisms due to its different chemical structure.

Researchers are now focusing on using purified individual ginsenosides to reveal the specific mechanism of action instead of using whole ginseng root extracts. The most commonly studied ginsenosides are Rb1, Re, Rg1, Rg3, and Rh1 [5]. The relevant ginsenosides (Rb1, Rg1, Rg3, and Rh1) for anticancer activity and corresponding molecular mechanisms are listed in Table 1.

Besides ginsenosides, ginseng polysaccharides also possess antitumor effect through modulation of innate immunity. Ginseng polysaccharides (present in 15% of ginseng root), including neutral and acidic polysaccharides, are water-soluble. It has been reported that* Panax* ginseng polysaccharides contain starch-like polysaccharide and pectin and can be fractionated into two neutral (WGPN and WGPA-N) and six acidic fractions (WGPA-1-RG, WGPA-2-RG, WGPA-1-HG, WGPA-2-HG, WGPA-3-HG, and WGPA-4-HG) by a combination of ethanol precipitation, ion exchange, and gel permeation chromatographies [57]. Many immunological studies have been performed with crude polysaccharide fractions, which are usually prepared by ethanol precipitation after extracting ginseng root with hot water. These polysaccharides have been reported to exert antitumor activity by regulating the immune response of the host organism. Using lymphocyte proliferation assays, both polysaccharides have been found to be potent B and T cell stimulators [57]. The acidic polysaccharides (10,000–150,000 MW), being readily soluble in water, are thought to be more active than neutral ones [27, 31, 32]. Ginseng pectin has also been shown to inhibit the actions of galectin-3, a **β**-galactoside-binding protein associated with cancer progression [58]. Nonsaponin constituents (immunomodulating polysaccharides) and the harmonizing constituents still remain to be explored.

## 4. Effects of Ginseng in Combination with Anticancer Drugs

### 4.1. *In Vitro* Studies

A number of* in vitro* studies have shown an enhanced anticancer effect when the ginseng extract or its active component is combined with a chemotherapeutic agent (see Table 2). One specific effect is increasing the cytotoxicity of chemotherapeutic agents.

Ginseng extracts, including ginsenosides, have been found to enhance the cytotoxicity of several chemotherapeutic agents such as 5-fluorouracil (5-FU, an antimetabolite), irinotecan (a plant alkaloid), mitomycin C (an antibiotics), docetaxel (a taxane agent belonging to a plant alkaloid), cisplatin (an alkylating agent), and others at the concentration range of 0.1–300 *μ*g/mL (see Table 2). One ginsenoside, Rg3, has been found to inhibit growth of various human tumor cells, such as prostate cancer cells (LNCaP, PC-3, and DU145), Lewis lung cancer cells, colon cancer cells (SW620 and HCT116), and B16 melanoma cells. Ginseng has been also found to enhance the cytotoxicity of docetaxel, cisplatin, and doxorubicin at low doses [21, 22]. Inhibition of nuclear factor-kappa (NF-**κ**B) may be one of the potential mechanisms of the observed effect. NF-**κ**B mediates tumor promotion, angiogenesis, metastasis, and resistance to chemotherapeutics through the expression of genes participating in malignant transformation and tumor promotion. Kim and coworkers have found that Rg3 can suppress the expression of several antiapoptosis genes (Bcl-2, Cox-2, c-Fos, c-Jun, cyclin D1, etc.) via inhibiting NF-**κ**B and thus enhancing the susceptibility of colon cancer cells to docetaxel and other chemotherapeutics [21, 22].

Also, panaxadiol, a pseudoaglycone of diol-type ginsenoside, has been found to enhance the anticancer effects of some anticancer drugs through the regulation of cell cycle transition and the induction of apoptotic cells. Apoptosis, highly regulatory process of programmed death involving the caspase protease family, is considered to be a key factor. Apoptosis may play an important role in the panaxadiol enhanced antiproliferative effects of irinotecan on human colorectal cancer cells as well as when used in combination with 5-FU [18, 20].

Furthermore, the synergistic inhibitory effect of* Panax *ginseng when combined with 5-FU has been observed in human gastric cancer cell line BGC823 [16]. This appears to involve NO which has been found to directly suppress the growth of BGC823 cells by inducing G0/G1 phase arrest through the regulation of Akt signaling pathway. Ginsenosides may increase NO production by inducing endothelial nitric oxide synthase (eNOS) phosphorylation via the ER-mediated PI3-kinase/Akt pathway [16].

One major difficulty in cancer chemotherapy is the development of broad anticancer drug resistance by tumor cells. This phenomenon has been termed multidrug resistance (MDR) [8]. The overexpression of P-glycoprotein (Pgp) or the multidrug resistance-associated proteins (MRPs) confer MDR to cancer cells. Ginseng extracts may induce chemosensitization of conventional anticancer agents via downregulation of MDR-1 expression (Pgp inhibition) [26, 28]. Choi et al. found that protopanaxatriol ginsenosides can potentially reverse Pgp-mediated MDR by increasing the intracellular accumulation of drugs through competitive inhibition of Pgp [26]. In addition, Shengmai can enhance the sensitivity of cancer cells (human lung carcinoma A549, gastric carcinoma SGC-7901, breast carcinoma MCF-7, and hepatocellular carcinoma HepG-2) to various anticancer drug such as gemcitabine (an antimetabolite), cisplatin, paclitaxel (a taxane agent belonging to a plant alkaloid), and epirubicin (an antibiotics) via downregulation of the mRNA level of MDR-1 [28].

Another benefit of ginseng when used with the anticancer drug is a potential reduction in drug induced toxicity. Baek et al. have found ginsenosides Rh4 and Rk3, the active principles of Sun ginseng (SG), to significantly reduce the cisplatin-induced nephrotoxicity in LLC-PK1 cells in a dose-dependent manner. The mechanisms of function and structure-activity relationships with other ginsenosides remain to be investigated [24]. Ginsenoside Rd may also ameliorate cisplatin-induced renal injury, a process in which apoptosis may play a central role [25].

A critical concern in the above* in vitro* studies is the relatively high concentration of ginseng extract/active components used (up to 300 *μ*g/mL). Such a high concentration is likely not achievable* in vivo*, as the *C*_max⁡⁡_ of some ginsenosides following oral administration in rats has shown to be less than 0.7 *μ*g/mL [59, 60]. Verification of* in vitro* benefit from lower concentrations of ginseng or from* in vivo* studies will be essential.

### 4.2. *In Vivo* Animal Studies

A number of positive benefits have been delineated with ginseng and anticancer drug combinations. They include increase of drug exposure, inhibition of the angiogenesis and metastasis, survival benefit, reduction of side effects of anticancer drugs, and therapeutic improvement (see Table 3).

A pharmacokinetic interaction leading to enhancement of certain anticancer drugs has been reported. After pretreatment with 3.0 mg/kg* Panax* ginseng extract orally twice daily for ten consecutive days, the elimination half-life of 5-FU has been shown to significantly increase by approximately 58.8% (79.17* versus* 125.72). The increase in *t*_1/2_ caused by* Panax* ginseng extract can result in a higher drug exposure of 5-FU, which may lead to a longer drug effect [16]. The specific mechanism however is not known. Ginseng may increase the exposure of other drugs including docetaxel.* In vitro* studies using human liver microsomes have suggested that ginseng as well as its various ginsenosides, at clinically relevant concentrations, can moderately inhibit CYP1A1, CYP1A2, CYP1B1, CYP2D6, CYP2C9, CYP2C19, CYP2E1, and CYP3A4 [61].

Angiogenesis, the process of pathological vascular in-growth critical for tumor expansion, is now known to play an important role in both growth and metastasis of some cancers. Ginsenoside Rg3 has been found to inhibit tumor angiogenesis. Combined therapy with Rg3 and low-dose gemcitabine or cyclophosphamide has been found to produce significant antiangiogenic effect without overt toxicity. The combined therapy has been shown to decrease vascular endothelial growth factor (VEGF) expression and microvascular density as well as blood flow in tumors (by color Doppler flow imaging) and peak systolic velocity when compared with the control mice. The combined therapy may have selectively enhanced the damage or cytotoxic effects of chemotherapy on newly formed blood vessels while simultaneously reduced Ki-67, VEGF, bcl-2, and P53 gene expression which may partially be responsible for their antiangiogenic and antitumor effects [11, 34, 36].

Survival benefit has been reported with the combination of ginseng or its active components with anticancer agents. In one study, combination treatment with paclitaxel (5 or 15 mg/kg) and acidic polysaccharide (25 mg/kg) has resulted in a 28.6 or 42.8% increase in 30-day life span of ICR mice bearing sarcoma 180 tumor cells, compared to paclitaxel treatment alone [27]. In another study, up to 53% of the BALB/c mice treated with combination of cyclophosphamide and an acidic polysaccharide (25 mg/kg) have shown an increase in survival rate compared with only 10% with cyclophosphamide alone [32]. At least 3 positive survival studies have been reported with ginsenoside Rg3 alone or in combination with anticancer drug: (1): treatment with Rg3, cyclophosphamide, or their combination in athymic mice bearing human ovarian cancer SKOV-3 has been found to improve survival 23.72, 25.90, and 27.12 days, respectively, compared to 13.6 days with the control [34]. (2): combination with gemcitabine has been found to increase survival rate (100%) compared with control or gemcitabine (60% or 70%) in 18 days after treatments [11]. (3): in 50% mice that survived cancer cell implantation, cyclophosphamide (low-dose), Rg3 alone, and their combination treatment groups result in 70, 77, and 95 days compared to only 29 days survival in the control group. The Rg3 anticaner drug combination treatment has shown to induce the longest survival [36]. The mechanism of such benefit however is not clear.

The combination of ginseng and various anticancer drugs have been found to lessen the reduction of weight loss, nausea/vomiting, diaphragm muscle toxicity, immunosuppression, and liver and renal function deterioration (see Table 3). Ginseng extract may decrease the side effect of weight loss from anticancer drugs by increasing the protein and RNA contents of muscles and liver in rats [11, 16, 31, 36, 62]. In addition, Ge and coworkers have found the effect of Shengmai (Chinese herbal preparation consisting of red ginseng, lilyturf root, and magnolia vine fruit) to be capable of protecting diaphragm muscles from doxorubicin induced toxicity which appears to be correlated with a decrease in expression of iNOS and lipid peroxidation [39]. Furthermore, Shengmai has been found to also protect liver and renal function and increase white blood cell, platelet counts, and serum alanine aminotransferase [38]. Also, ginseng is known to modulate the immune system and thus may improve chemotherapy by an “indirect effect.” Shim et al. have shown Korean ginseng can increase the expression level of the cytokines, such as TNF-*α*, IL-1*β*, IL-6, SCF and, GM-CSF [32]. Polysaccharides may also improve drug-induced immunosuppression. They can significantly increase relative spleen weight (spleen weight/100 g of bodyweight, e.g., frm 2.89% cyclophosphamide alone to 3.42% combined use), stimulate lymphocyte proliferation, NK cell cytotoxicity, and macrophage activity, increase serum TNF-*α*, IL-12, IFN-*γ*, and CRP (serum C-reactive protein) levels, and so forth [27, 31, 33]. However whether the degree of elevation of these cytokines by ginseng can improve chemotherapy outcome needs further investigated.

### 4.3. Clinical Studies

Despite popular use of ginseng, only a limited number of clinical studies have been reported on ginseng—chemotherapeutic agent combination (see Table 4). On the basis of the traditional Chinese medicine (TCM) consideration, the Chinese ginseng preparation Shengmai is selected as a tonic in combination with chemotherapy, for example, supplementing qi (means vital energy) and nourishing yin (means passive force) [40]. Chen et al. have evaluated the efficacy and side effects of Shengmai combined with chemotherapeutic agents in treating advanced non-small-cell lung cancer (NSCLC) [40]. This study was conducted on 63 patients with stages III B and IV NSCLC receiving navelbine (trade name of Vinorelbine, a plant alkaloid) and cisplatin chemotherapy. The patients were assigned to two groups: 33 patients in the treatment group receiving Shengmai by intravenous drip and Gujin Granule (a Chinese herbal remedy in water soluble granule) orally and 30 patients in the control group. Among the 61 patients (33 from the treatment group and 28 from the control group) who completed the observation, the response rate was 48.5% (16/33) in the treatment and 32.2% (9/28) in the control groups, with a median survival time of 13 months and 9 months, respectively. This study indicated that the combined use of ginseng and anticancer drug might enhance the short-term therapeutic efficacy of NSCLC. This study however was not blinded. A randomized, double-blind, placebo-controlled trial evaluating the therapeutic efficacy of Shenmai (same as Shengmai here) in cancer patients undergoing chemotherapy or radiotherapy is ongoing [43].

In another randomized controlled trial, Huang et al. evaluated the efficacy of Shenyi (95% ginsenoside Rg3) in combination with gemcitabine plus cisplatin in 60 patients with advanced esophageal cancer. Compared to patients in the control group with chemotherapy alone, the results showed no significant difference in total response rate between the two groups during the treatment phase. After treatment, the vascular endothelial growth factor in the treatment group was found to be lower than that in the control group (*P* < 0.05), suggesting an effect of inhibiting angiogenesis. In addition, one-year survival rate in the treatment group was higher compared with the control group (*P* < 0.05). Shenyi also improved the patients' quality of life according to the Karnofsky performance status scale [46]. Rg3 had been shown to have some anticancer activities like antiproliferative, apoptotic, antiangiogenic, antimetastatic, and anti-invasive effects as well as cell cycle regulation [8, 11, 12].

## 5. Summary and Conclusion

Ginseng has been used primarily as a tonic to benefit cancer patients, especially in Asia. Based on our review of published* in vitro*,* in vivo,* and human studies, ginseng has excellent potential as a chemotherapy adjuvant, because of its low toxicity and many desirable properties such as antiangiogenesis, antiproliferation, anti-inflammation, antioxidation, apoptosis, and immune modulation effects [63]. Although there are substantial evidence from* in vitro* and animal studies showing the benefit of ginseng and its active constituents in enhancing antitumor activity when used in combination with other anticancer drugs, there is insufficient clinical evidence of such benefit at present.

Ginseng has already been accepted as a natural product for health promotion. For this reason, continued use of ginseng together with encouraging results from the* in vitro* and* in vivo* animal studies (see Tables 2 and 3) may provide important clues to demonstrate future clinic benefit of ginseng. Further studies of ginseng products should include quality control such as the use of activity markers and active components, as well as determination of their pharmacokinetics and pharmacodynamics. It is hoped that government support as well as development of new process patents for ginseng will provide sufficient incentive and funding to conduct well designed clinical trials leading to regulatory approval of a ginseng product for chemotherapy enhancement in the future.

## Figures and Tables

**Table 1:** Anticancer activities of commonly studied relevant ginsenosides [5, 8, 11–14].

Ginsenoside	Anticancer activity	Molecular mechanism
Rb1	Weakly antiproliferative; antiangiogenic	(i) Inhibit capillary genesis (ii) Inhibit TNF-a release (iii) Protect against oxidative stress (iv) Inhibit tube-like structure formation of endothelial cells by regulating pigment epithelium-derived factor (PEDF) through estrogen receptor-**β** [13, 14]
Rb3	No antiproliferative activity	Inhibit TNF-*α* release
Rg1	Antiproliferative	(i) Inhibit oncogenes c-myc, c-fos (ii) Downregulate nucleophosmin.
Rg3	Antiproliferative, apoptotic, antiangiogenic, antimetastatic, anti-invasive, and cell cycle regulation, [8, 11, 12]	(i) Regulate mitochondrial cytochrome C, poly ADP ribose polymerase (PARP) and C9 (ii) Inhibit MMP-2 and 9 (iii) Inhibit adhesion of metastatic cells to basement membrane (iv) Inhibit MDR (most potent among all ginsenosides)
Rh1	Causes differentiation of teratocarcinoma cells, strongly apoptotic	(i) Bind to steroid receptor (ii) Inhibit TNF-*α* (iii) Inhibit phosphorylation of JAK1, STAT1, STAT3, and ERK

**Table 2:** *In  vitro* studies of ginseng in combination with other anticancer drugs.

Ginseng products	Source	Cells	Conc.	Anticancer drugs	Direct action (cytotoxicity)	Indirect action	Reference
*Panax * ginseng extracts (RG and WG)	Tongrentang Pharmacy, Beijing, China; WG compared with RG: lower in Rg1, Rb1 and Rd; higher in Rg3.	HCT-116	100~300 *μ*g/mL	5-FU	HCT-116 (+): RG > WG	(1) Apoptosis induction: RG (−) (2) Cell cycle arrest: RG (G1 phase)	[15]

*Panax* ginseng extracts	Tongrentang Pharmacy, Beijing, China, major ginsenosides Rg1, Rb1, Rd	BIU-87, A549, SW480, BGC823	0.1–100 *μ*g/mL	5-FU	BGC823 (+) SW480 (−) A549 (−) BIU-873 (−)	None	[16]

Ginsenoside Panaxadiol	National Institute for the Control of Pharmaceutical and Biological Products, Beijing, China	HCT-116, SW-480	10 *μ*M	Irinotecan	HCT-116 (+) SW-480 (+)	(1) Apoptosis induction (2) Cell cycle arrest: G1 phase [17]/S phase [14]	[18]
		HCT-116	5~25 *μ*M	5-FU	HCT-116 (+)		[19]

Ginsenoside Panaxytriol	Red *Panax* ginseng, Nikkan Korai Ninjin, Kobe, Japan	MK-1	1~12.5 *μ*g/mL	mitomycin C	MK-1 (+)	Increase cellular drug accumulation (by decreasing membrane fluidity)	[20]

Ginsenoside Rg3	Korea ginseng: Sun ginseng	SW620, HCT116	25~100 *μ*M	Docetaxel cisplatin doxorubicin paclitaxel	SW620 (+) HCT116 (+)	(1) Apoptosis induction (2) Cell cycle arrest: G0/G1 phase (3) Decrease drug resistant by inactivating NF-kappaB	[21]
		LNCaP, PC-3, DU145		Docetaxel cisplatin doxorubicin	LNCaP (+) PC-3 (+) DU145 (+)		[22]

Ginsenosides	Red *Panax* Ginseng	Leukemic progenitor cells	20 *μ*g/mL	Homoharringtonine cytarabine, adriamycin, and etoposide	Leukemic progenitor cells (+)	Stimulate progenitor cell proliferation by driving noncycling progenitors to enter cell cycle	[23]

Ginsenosides, Rh4 and Rk3 (unique ginsenosides of SG/RG)	Korean ginseng: WG: Korea local SG and RG: Ginseng Science Inc. Seoul, Korea	LLC-PK_1_	10~160 *μ*g/mL, Rh4/Rk3: 5~20 *μ*g/mL	Cisplatin	N/A	Reduce drug-induced renal injury: (i) increase cell viability: RG/Rh4/Rk3 (+), WG (−) (ii) decrease LDH leakage: Rh4/Rk3 (+)	[24]

Ginsenoside Rd	Self-prepared from Korean ginseng	LLC-PK_1_	25~125 *μ*M	cisplatin	N/A	Reduce drug-induced renal toxicity: (i) Decrease LDH leakage (ii) Suppress apoptosis	[25]

Ginsenosides, protopanaxatriol ginsenosides (major Rg1, Re), protopanaxadiol ginsenosides (major Rb1, Rb2, and Rc)	Korean red ginseng: Korea Ginseng and Tobacco Research Institute, Taejeon, Korea	AML-2/D100 (overexpress Pgp), AML-2/DX100 (overexpress MRP)	50~300 ug/mL	Daunorubicin	AML-2/D100: PTG (+), others (−)	Decrease drug resistant: inhibit Pgp activity (protopanaxatriol group)	[26]

Acidic polysaccharide	Korean red ginseng: Korea Ginseng Cooperation, Daejeon, Korea	BALB/C mouse splenocytes and macrophages	10–1000 *μ*g/mL	paclitaxel	N/A	Reduce drug-induced toxicity (immunosuppression): (i) restore splenocyte proliferation; (ii) increase macrophage cytotoxicity	[27]

Shengmai (Chinese herbal preparation consisting red ginseng, lilyturf root, and magnolia vine fruit)	China, no detailed description	A549, SGC-7901, MCF-7, HepG-2	30 *μ*g/mL	gemcitabine, cisplatin, paclitaxel, and epirubicin	A549 (+) SGC-7901 (+) MCF-7 (+) HepG-2 (+)	Decrease drug resistant: downregulating mRNA expression level of MDR-1.	[28]

Human bladder cancer cell line (BIU-87); human lung cancer cell line (A549); human colon cancer cell line (SW480); human gastric cancer cell line (BGC823, MK-1, and SGC-7901); human colorectal cancer cells (HCT-116, SW-480, and SW620); human prostate cancer cell lines (LNCaP, PC-3, DU145); human breast carcinoma (MCF-7), human hepatocellular carcinoma (HepG-2), pig renal tubular epithelial cells (LLC-PK_1_), two resistant acute myelogenous leukemia (AML) sublines: daunorubicin- and doxorubicin-resistant AML-2 subline (AML-2/D100 and AML-2/DX100 overexpress Pgp and MRP, respectively); red ginseng (RG), white ginseng (WG); Sun ginseng (SG); p-glycoprotein (Pgp); multidrug resistance-associated protein (MRP); “+”: positive; “−”: negative.

**Table 3:** *In  vivo* studies of ginseng in combination with other anticancer drugs.

Ginseng products	Source	Animals	Dose	Anticancer drug	Direct action (inhibit tumor growth)	Indirect action	Reference
*Panax* ginseng extracts	Tongrentang Pharmacy, Beijing, China, major Rg1, Rb1, Rd	Rat	3.0 mg/kg po., bid, 10 days	5-FU	N/A	(1) Increase drug elimination half-life: *t*_1/2_(*k*_*e*_) of control and ginseng treated group 79.17 and 125.72 min, respectively (*P* < 0.05) (2) Reduce drug-induced weight loss;	[16]
	Korean ginseng, Ginseng Nonghyup, Keum-san, Korea	Rat	12.5~100 mg/kg, po.,	cisplatin	N/A	Reduce drug-induced nausea and vomiting	[29]
	Korean red ginseng, Korea Ginseng Cooperation, Daejeon, Korea	Ferret	3 g/kg, po.,				[30]

acidic polysaccharides	Korean red ginseng, Korea Ginseng Cooperation, Daejeon, Korea	ICR mice bearing sarcoma 180; C57BL/6 mice bearing B16 melanoma	25, and 100 mg/kg, ip., 7 days	paclitaxel	Yes	(1) Improve survival rate (ICR mice bearing sarcoma 180): 28.6 and 42.8% increase in 30-day life-span, while no obvious effect seen on drug-treatment alone. (2) Reduce drug-induced immunosuppression: increase NK cell cytotoxicity (C57BL/6 mice)	[27]
	Korean red ginseng, Korean Tobacco, and Ginseng company	BALB/c mice	33~300 mg/kg po., 3 wks	Cyclophosphamide (CP)	N/A	Reduce drug-induced: (1) weight loss; (2) immunosuppression: (i) increase spleen weight (ii) restore splenocyte proliferation (iii) increase macrophage activity (NO production) (iv) increase NK cell cytotoxicity (v) increase serum IL-12, IFN-*γ* and CRP (C-reactive protein) level	[31]
	Korean ginseng	BALB/c mice: C57BL/6 mice bearing mouse lung carcinoma LLC cells	100 mg/kg, i.p.	Cyclophosphamide (CP)	Yes	(1) Improve survival rate (BALB/c mice): 53% of post-treated group increased in the 30-day life-span compared with only 10% in the drug alone treated group. (2) Reduce drug-induced immunosuppression. (i) Accelerate recovery of bone marrow cells and blood neutrophils (ii) Stimulate splenocyte proliferation and maintain its cytotoxicity. (iii) Increase cytokine mRNA expression (TNF-*α*, IL-1*β*, IL-6, SCF and GM-CSF).	[32]

neutral polysaccharides	*Panax* ginseng, Changbai Mountain, Jilin, China	ICR mice bearing Sarcoma 180	25~150 mg/kg, po., 10 days	5-FU	Yes	Reduce drug-induced immunosuppression: (i) increase spleen weight, (ii) stimulate lymphocyte proliferation, (iii) increase NK cell cytotoxicity, (iv) enhance macrophage activity (phagocytosis and NO production), (v) increase serum TNF-a level	[33]

Ginsenoside Rg3	American ginseng; ≥99.5%, provided by Department of Medicinal Chemistry of Preclinical Medicine of Jilin University, China	Athymic mice bearing human ovarian cancer SKOV-3	3.0 mg/kg, ip., 10 days	Cyclophosphamide (CP)			[34]
	*Panax* red ginseng, from Northeast China, ≥99.5%, YaTai Pharmaceutical Company, China	C57/BL6 mice with Lewis lung carcinoma	20 mg/kg, po. 18 days	gemcitabine	Yes: (1) Decrease tumor weight (2) Inhibit tumor cell proliferation [19] (3) Increase tumor necrosis rate [20]	(1) Improve survival: (i) Rg3, CP, and combination group had longer survival (23.72, 25.90, and 27.12 days, respectively) compared with control group (13.61 days, *P* < 0.05) (*n* = 7) [19]; (ii) 18 days after treatments, combination group resulted in increased survival rate (100%) compared with control or gemcitabine group (60% or 70%, *P* < 0.05) (*n* = 10) [20] (iii) Control group, low-dose CP or Rg3 alone group, and combination group had 29, 70 or 77, and 95 days of 50% survival rate from implantation, respectively. (*n* = 20) [35] (2) Improve life quality (3) Reduce drug-induced: weight loss, leucopenia, limited motility, and skin discoloration. (4) Inhibit tumor angiogenesis: (i) decrease microvascular density (ii) decrease vascular endothelial growth factor expression	[11]
			10 mg/kg, po. 21 days	Cyclophosphamide (CP)			[36]

Ginsenoside Rg1	*Panax* ginseng, provided by Takeda Chemical Industries, Osaka.	BALB/c mice	10 mg/kg, i.p. 3 days	Cyclophosphamide	N/A	Reduce drug-induced immunosuppression	[37]

Ginsenoside Rd	*Panax* ginseng	Rat	1 and 5 mg/kg, po. 30 days	cisplatin		Reduce drug-induced renal toxicity	[23]

Shengmai (Chinese herbal preparation consisting red ginseng, lilyturf root and magnolia vine fruit)	Jilin Province Ji'an Yisheng Pharmaceutical Co. Ltd., China	Rat	3 mL/kg, i.p., pre and during treatment, 4 wks	doxorubicin (DOX)	N/A	Reduce drug-induced diaphragm muscle toxicity	[38]
	Shanghai Hutchison Pharmaceuticals	Mice bearing hepatoma	3.5~14 mL/kg/d, 14 days	5-FU	Yes	(1) Improve immunological function (2) Reduce drug induced adverse reaction: (i) protective effect on liver and renal function (ii) increase WBC and PLT counts	[39]

**Table 4:** Clinical studies of ginseng in combination with other anticancer drugs.

Ginseng products	Source	Study design	Anticancer drug	Cancer type	Endpoints and results	Reference
Shengmai (Chinese herbal preparation consisting of red ginseng, lilyturf root, and magnolia vine fruits)	Ya, an Sanjiu Pharmaceutical Co., Ltd, China	Randomized controlled open design, two groups: (1) control group (*n* = 28): anticancer drugs alone, (2) treatment group (*n* = 33): anticancer drugs + Shengmai 100 mL/day, 14 days + Gujin Granule (a Chinese herbal remedy) 10 g, t.i.d., po. 6 months	Navelbine + cisplatin	Non-small-cell lung cancer	(i) Response rate: 48.5% (16/33) in treatment versus 32.2% (9/28) in control groups, *P* < 0.05; (ii) median survival time: 13-month treatment group versus 9-month control group, *P* < 0.05; (iii) 1-year survival rate: NS (iv) median time to progression: NS (v) hematological toxicity: NS (vi) cycles of chemotherapy: NS	[40]
	China, no detailed description	Two groups: (1) routine group (*n* = 56): infuse 80 mL Shengmai alone after chemotherapy; (2) improved group (*n* = 75): infuse Shengmai 40 mL before and after chemotherapy, respectively	No detailed description	No detailed description	Chemotherapy induced phlebitis: *P* < 0.01 (incidence 20% in improved group, 50% in routine group)	[41]
		Two groups: (1) control group (*n* = 26): anticancer drugs alone, 21-day/cycle, 2 cycles; (2) test group (*n* = 33): anticancer drugs + infuse Shengmai 50 mL 14-day/cycle, 2 cycles	Etoposide + folinic acid + 5-FU	Advanced gastric cancer	(i) Life quality improvement: *P* < 0.05 (ii) Reduce drug induced: *P* < 0.05 (iii) Gastrointestinal effect (iv) Myelosuppression	[42]
	Sen-Ten Pharmaceutical Company, Taiwan	Randomized, double-blind, two groups: (1) test group, (2) placebo-control group, 4 weeks	No detailed description	No detailed description	Antifatigue activity (ongoing)	[43]

Ginsenoside Rg3	YaTai Pharmaceutical Company, China	Two groups: (1) control group (*n* = 30): anticancer drugs + Rg3 capsule; (2) test group (*n* = 61): anticancer drugs + Rg3 table	No detailed description	Breast cancer	Immunoimprovement: (i) increase level of T cell subtype function (CD4/CD8), *P* < 0.001 (ii) improve symptoms of Q-deficiency according TCM, *P* < 0.01 or *P* < 0.05	[44]
		Randomized, prospective, multicenter, two groups: (1) placebo group (*n* = 61) (2) test group (*n* = 54): Rg3 capsule, po, b.i.d.,	vinorelbine + cisplatin	Advanced non-small-cell lung cancer	(i) Improve response rate: *P* < 0.05 (ii) Improve survival time: 9.7 months (mean) and 8.0 months (median) in placebo group; 15.3 months (mean) and 10.0 month (median) in test group; *P* < 0.01 (iii) Weight, general conditions, and adverb reactions: *P* > 0.05	[45]
		Randomized controlled trial, two groups: (1) control group (*n* = 30) (2) treatment group (*n* = 30): Rg3 capsule, po, 20 mg, b.i.d., 30 days	Gemcitabine + cisplatin	Advanced esophageal cancer	(i) Improve response rate: NS (ii) Improve quality of life: *P* < 0.05 (iii) Increase 1-year survival rate: *P* < 0.05 (iv) Reduce drug induced adverse reaction: nausea and vomiting, WBC and PLT counts; *P* < 0.05	[46]
